# Dissociated response to PD-1 inhibitors combined with radiotherapy in patients with advanced metastatic solid tumors: a single-center experience

**DOI:** 10.1186/s12957-023-03122-6

**Published:** 2023-07-27

**Authors:** Qin Yu, Haiyan Zhang, Yan Song, Chen Chen, Jin Chen, Junkang Shen

**Affiliations:** 1 Department of Radiology, The Second Affiliated Hospital of Soochow University, Suzhou, China; 2 Department of Imaging, Jiangsu Vocational College of Medicine Affiliated Dongtai People’s Hospital, Kangfu West Road 2, Dongtai, Jiangsu Province 224000 China; 3 Department of Pathology, the Third People’s Hospital of Nantong, Nantong, China; 4 Department of Pathology, Affiliated Nantong Hospital 3 of Nantong University, Nantong, China; 5 Department of Radiology, Jieshou City People’s Hospital, Fuyang, China; 6 Department of Orthopedics, Jiangsu Vocational College of Medicine Affiliated Dongtai People’s Hospital, Kangfu West Road 2, Dongtai, 224200 China; 7 Department of Orthopedics, Dongtai People’s Hospital, Kangfu West Road 2, Dongtai, 224000 Jiangsu Province China; 8 Institute of Imaging Medicine, Soochow University, Suzhou, China; 9 Department of Imaging, The Second Affiliated Hospital of Soochow University, No 1055 Sanxiang Road, Soochow, 215000 Jiangsu Province China

**Keywords:** Advanced metastatic solid tumor, Anti-programmed death 1 (PD-1), Radiotherapy (RT), Dissociated response (DR), Overall survival (OS)

## Abstract

**Background:**

Anti-programmed death 1/anti-programmed death ligand 1 (PD-1/PD-L1) combined with radiotherapy (RT) has a synergistic effect on systemic tumor control. A dissociated response (DR), characterized by some lesions shrinking and others growing, has been recognized with immune checkpoint inhibitor (ICI) monotherapy or combination therapy. The objective of this study was to assess the frequency and clinical benefit of DR in patients with advanced metastatic solid tumors receiving PD-1 inhibitors in combination with RT.

**Methods:**

We conducted a single-center retrospective analysis of patients with advanced metastatic solid tumors receiving PD-1 inhibitor combined with RT at the Department of Radiotherapy & Oncology, The Second People’s Hospital Affiliated with Soochow University. Treatment response was assessed for each measurable lesion according to the Response Evaluation Criteria in Solid Tumours ( RECIST) v 1.1 guidelines. Patterns of response are divided into four groups: (1) DR, (2) uniform response, (3) uniform progression, and (4) only stable lesions. The overall survival (OS) of different groups was compared using Kaplan–Meier methods and log-rank tests.

**Results:**

Between March 2019 and July 2022, 93 patients were included. The median follow-up was 10.5 months (95% CI 8.8–12.1). The most common tumor types were lung cancer (19.8%), colorectal adenocarcinoma (17.2%), and esophageal cancer (10.8%). DR was observed in 22 (23.7%) patients. The uniform progression and DR are two different patterns of progression. After confirming progression, the overall survival of patients with DR was significantly longer than that of patients with uniform progression (9.9 months (95%CI 5.7-14.1) vs. 4.2 months (95%CI 1.9-6.5), *P* = 0.028). Compared with DR patients who did not continue PD-1 inhibitor combined with RT or PD-1 inhibitor monotherapy (*n* = 12), DR patients who continued treatment (*n* = 10) had significantly longer OS (15.7 (95%CI 3.5-27.9) vs 8.2 (95%CI 5.6-10.8) months, *P* = 0.035).

**Conclusions:**

DR is not uncommon (23.7%) in patients with advanced metastatic solid tumors treated with PD-1 inhibitors combined with RT and shows a relatively favorable prognosis. Some patients with DR may benefit from continued PD-1 inhibitor therapy in combination with RT or PD-1 inhibitor monotherapy and may have longer OS.

## Introduction

In recent years, based on the breakthrough of tumor immunobiology, the development of tumor immunotherapy has opened a new chapter for the treatment of malignant tumors. Immunotherapy does not target the tumor cells themselves, but overcomes the immunosuppression caused by the tumor and its microenvironment, enhances the immunogenicity of tumor antigens, stimulates and improves anti-tumor immune response, and enables the immune system to target and kill cancer cells [1, 2]. Immune checkpoint inhibitors (ICIs), including anti-cytotoxic T-lymphocyte antigen-4 (CTLA-4), PD-1, and PD-L1 antibodies, have dramatically changed the paradigm of oncology treatment and its assessment, achieving significant therapeutic outcomes in major types of advanced solid tumors [3]. However, many patients do not respond or respond only briefly to PD-1/PD-L1 monotherapies due to immunosuppressive factors in their bodies [4–7]. There is growing evidence that radiotherapy (RT) can both stimulate local and systemic immunostimulatory effects, which can be synergistic with immunotherapy in systemic tumor control. More and more clinical studies have demonstrated the potential synergistic effect of PD-1/PD-L1 blocking therapy and radiotherapy in patients with advanced cancer [8–10].

Due to ICIs’ unique mechanism of action [11], the tumor response pattern of ICI monotherapy or combination therapy may be different from that of conventional cytotoxic chemotherapy or targeted therapy (remission, stabilization, progression), such as pseudo-progressive disease (PsPD), DR, delayed response (DeR), hyperprogressive disease (HPD), and more recently fast progressive disease (FastPD) [12–18]. These atypical radiological response patterns do not fully conform to the RECIST v 1.1 guidelines and are relevant to clinical treatment decisions. DR is simply defined as an increase in the size of some lesions with shrinkage of others [19]. In recent years, the definition of DR is more detailed and specific, but there is still a lack of unity. This atypical response confused stopping or continuing ICI therapy.

To date, there is limited data on the incidence of DR in ICIs combined with RT, and its clinical significance is not fully understood [20]. Therefore, in this study, we aim to evaluate the occurrence of DR in patients with advanced metastatic solid tumors treated with PD-1 inhibitors combined with radiotherapy and its correlation with prognosis, to provide a reference for clinical treatment.

## Materials and methods

### Patients

We retrospectively analyzed patients with advanced metastatic solid tumors who received PD-1 inhibitors combined with RT in the Department of Radiotherapy & Oncology, The Second People’s Hospital Affiliated with Soochow University, from March 2019 to July 2022. The cutoff date for data collection was December 31, 2022. All patients were given stereotactic body radiotherapy (SBRT) or hypofractionated radiotherapy (HFRT; 5 Gy or 8 Gy *3), and anti-PD-1 antibody (200 mg per body) was injected intravenously within 1 week after radiotherapy. Clinically assessed patients with the stable disease continue to be treated with PD-1 inhibitors as maintenance monotherapy until clinical or radiological disease progression or unacceptable toxicity. Inclusion criteria are (1) patients with advanced metastatic solid tumor and age ≥ 18 years and (2) the score of the Eastern Cooperative Oncology Group performance status (ECOG ps) ≤ 3. Exclusion criteria are (1) combined with other therapies, including chemotherapy, targeted therapy, antiangiogenic therapy, etc.; (2) CT/MRI was not available at baseline (within 28 days before treatment) and follow-up to evaluate efficacy; (3) without measurable lesions at baseline; and (4) receiving PD-1 inhibitor combined with RT < 2 cycles. This study was approved by the Medical Ethics Committee of the hospital. Due to the retrospective study design, no written informed consent was required.

### Clinical variables

Patients’ age, gender, primary tumor, ECOG status, number of previous systemic treatments, and number of organs involved in metastasis were collected from the electronic medical record (EMR). OS was defined as the time from the start of the first cycle of the PD-1 inhibitors combined with RT to death or the last follow-up. A durable clinical benefit was defined as treatment continuation over 6 months [21].

### Tumor assessment

All patients were assessed every 6–8 weeks, and the relative diameter change of each non-irradiated measurable lesion between baseline and follow-up was evaluated. According to RECIST 1.1 criteria, measurable lesions were defined as non-lymph node lesions ≥ 10 mm in the long axis and lymph node lesions ≥ 15 mm in the short axis. A responding lesion was defined by a decrease in lesion size of > 30%. A progressive lesion was defined by an increase in lesion size of > 20% and the diameter increased by at least 5 mm. Patterns of tumor response are divided into four groups: (1) DR: ① the presence of both progressive and responding lesions and ② only responding lesions, but new lesions or unmeasurable lesions significantly worsened (irrespective of stable lesions); (2) uniform response: only responding lesions with no new lesions or unmeasurable lesions with significant deterioration (irrespective of stable lesions); (3) uniform progression: only progressive lesions (irrespective of stable lesions); and (4) only stable lesions. All radiological images were evaluated independently by two radiologists (8 and 14 years of experience in oncologic imaging), and in cases of disagreement, images were re-examined until a consensus was reached.

### Statistical analysis

IBM SPSS (Armonk, New York) statistics version 26.0 was used for statistical analyses. Continuous variables with normal distribution were represented by mean ± standard deviation and analyzed by the Student’s *t*-test. Categorical variables were described by *n* (%) and compared using chi-square tests or Fisher exact tests. OS were generated using the Kaplan–Meier method and compared using a log-rank test. All statistical tests were two-sided, and *P* values < 0.05 were considered statistically significant.

## Results

### Patient characteristics

**Fig. 1:**
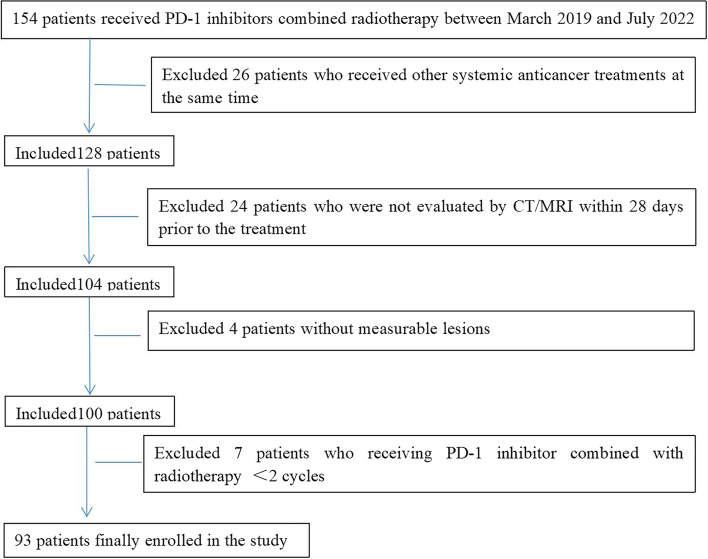
Patient selection flow

A total of 93 patients were enrolled (Fig. 1). The median follow-up was 10.5 months (95% CI 8.8–12.1). The mean age is 63 years (range: 31–87), 53.8% (50/93) were male, 78.5% (73/93) had ECOG PS of 2 or 3, 22.6% (21/93) had received 3 or more systemic therapies before treatment, and 26.9% (25/93) had 3 or more metastatic organs. Primary tumor types are lung cancer (19.8%), colorectal adenocarcinoma (17.2%), esophageal cancer (10.8%), stomach (7.5%), and cervical cancer (7.5%). 81.8% of patients had one or two irradiated sites (range: 1–5). 29.0% (27/93) showed a uniform response, 23.7% (22/93) showed DR, 25.8% (24/93) showed uniform progression, and 21.5 (20/93) presented only stable lesions. Patients’ characteristics are summarized in Table 1.

**Table 1:** Patient clinical characteristics

Characteristic	Total(%) *n* = 93	DR *n* = 22	No-DR *n* = 71	*P* value
Age^*^	63±13	60±13	64±12	0.243
Sex				
Female	43 (46.2)	15 (68.2)	28 (39.4)	0.018
Male	50 (53.8)	7 (31.8)	43 (60.6)	
ECOG ps				
1	20 (21.5)	3 (13.6)	17 (24.0)	0.566
2	52 (55.9)	14 (63.7)	38 (53.5)	
3	21 (22.6)	5 (22.7)	16 (22.5)	
No. of prior systemic therapies				
< 3	72 (77.4)	15 (68.2)	53 (74.6)	0.550
≥ 3	21 (22.6)	7 (31.8)	18 (25.4)	
Metastatic organs involved				
< 3	68 (73.1)	16 (72.7)	56 (78.9)	0.547
≥ 3	25 (26.9)	6 (27.3)	15 (21.1)	
PD-L1 status				0.260
< 1	15 (16.1)	5 (22.7)	10 (14.1)	
≥ 1	19 (20.4)	2 (9.1)	17 (23.9)	
Unknown	59 (63.5)	15 (68.2)	44 (62.0)	
Primary cancer sites				0.541
Lung	18 (19.4)	4 (18.2)	14 (19.7)	
Colorectum	16 (17.2)	3 (13.7)	13 (18.3)	
Breast	4 (4.3)	1 (4.5)	3 (4.2)	
Gastro	7 (7.5)	0 (0.0)	7 (9.9)	
Cervix	7 (7.5)	4 (18.2)	3 (4.2)	
Esophagus	10 (10.8)	2 (9.1)	8 (11.3)	
Ovary	5 (5.4)	2 (9.1)	3 (4.2)	
Head and neck	6 (6.5)	1 (4.5)	5 (7.0)	
Liver	6 (6.5)	1 (4.5)	5 (7.0)	
Pancreas	6 (6.5)	1 (4.5)	5 (7.0)	
Others	8 (8.4)	3 (13.7)	5 (7.0)	
No. of irradiated sites				
1	38 (40.9)	6 (27.3)	32 (45.1)	0.332
2	38 (40.9)	11 (50.0)	27 (38.0)	
≥ 3	17 (18.2)	5 (22.7)	12 (16.9)	
Tumor response by lesion to lesion				
Uniform response	27 (29.0)			
Dissociated response	22 (23.7)			
Only stable lesions	20 (21.5)			
Uniform progression	24 (25.8)			

*ECOG ps* ECOG PS Eastern Cooperative Oncology Group Performance Status

^*^Mean ± standard deviation

### Dissociated response

**Fig. 2:**
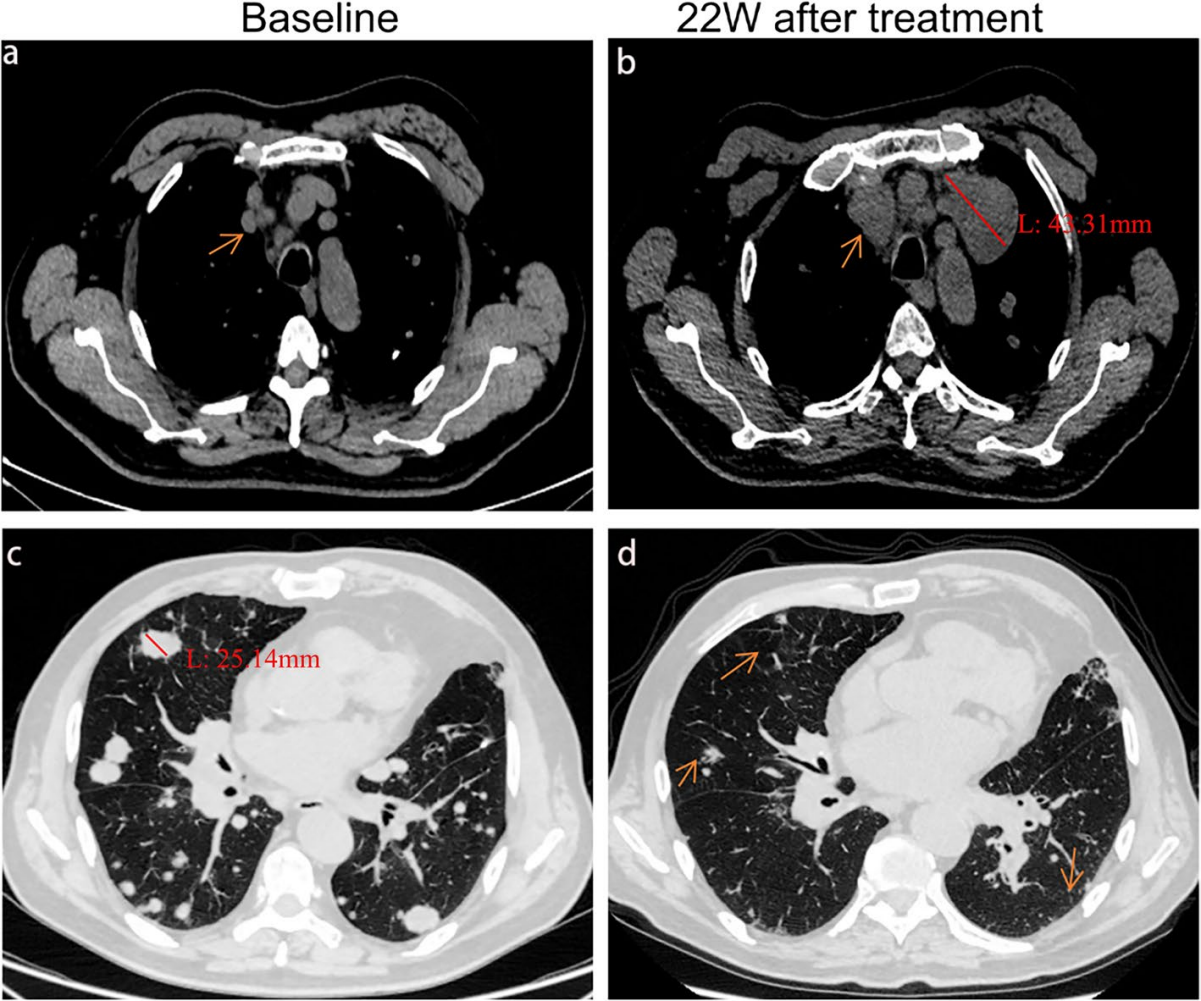
Representative radiological data of patients with dissociated responses. A 67-year-old man with advanced lung adenocarcinoma. He started PD-1 inhibitors combined with radiotherapy as a fifth-line therapy. The radiation site was a metastatic lesion of the right iliac crest. After 22 weeks of treatment, his pulmonary metastasis lesions (the largest: 25 mm) reduced in size (**c**, **d**), although two new metastatic lymph nodes (the larger: 43 mm) in the mediastinum were detected on the CT scan (**a**, **b**); therefore, DR was diagnosed

DR with the combination of PD-1 inhibitors and radiotherapy occurred in 23.70% (22/93) of patients (Fig. 2). Female patients in the DR were significantly more than those in the non-DR group (*P* = 0.018), and there was no significant difference in other clinical characteristics (Table 1). DR appeared between 7 and 38 weeks after the start of treatment. At DR diagnosis, 9.1% (2/22) patients showed RECIST-defined PR, 54.5% (12/22) patients showed RECIST-defined SD, and 36.4% (8/22) patients showed RECIST-defined PD. 54.5% (12 /22) of DR patients chose to continue PD-1 inhibitor combined with radiotherapy or PD-1 inhibitor monotherapy, of which 11 patients achieved a durable clinical benefit. Characteristics of DR patients are represented in Table 2.

**Table 2:** Characteristics of the patients who exhibited an DR

No.	Gender	Age (years)	Histology (tumour type)	Time of DRAppears (weeks)	RECIST 1.1	Response site	Progression site	ICIs treatment after DR (months)	Duration of ICIs treatment after DR (months)
1	F	67	Adenocarcinoma (lung)	8	SD	liver	lung	PD-1+RT	13.5
2	M	76	Adenocarcinoma (colorectum)	21	SD	liver	liver	None	/
3	M	67	Adenocarcinoma (lung)	22	SD	lung	lung, LN	None	/
4	F	75	Adenocarcinoma (lung)	7	SD	peritoneal cavity	colon	PD-1	15.7
5	F	58	Sarcoma (retroperitoneum)	23	SD	peritoneal cavity	peritoneal cavity	PD-1	13.5
6	F	64	Infiltrating ductal carcinoma (Breast)	21	PR	lung	lung	PD-1	26
7	F	65	Leiomyosarcoma (Cervix)	7	SD	lung, rectum	lung	PD-1+RT	11.5
8	F	53	Papillar carcinoma (kidney)	8	SD	LN	LN	PD-1+RT	11.9
9	M	68	Squamous cell carcinoma (lung)	15	PD	LN	NL (lung)	PD-1+RT	19.4
10	F	53	Squamous cell carcinoma (lung)	24	PD	peritoneum, pleura	pleura	None	/
11	M	62	Squamous cell carcinoma (esophagus)	12	SD	lung	lung	None	/
12	M	77	Adenocarcinoma (colorectum)	8	PD	lung, liver	lung, liver	PD-1+RT	3.4
13	F	52	Adenocarcinoma (cervix)	16	SD	liver	LN	None	/
14	F	76	Adenocarcinoma (bile duct)	12	PD	liver	NL (liver)	None	/
15	M	71	Adenocarcinoma (prostate)	38	PD	lung	lung	None	/
16	F	51	Serous epithelial carcinoma (ovary)	13	SD	peritoneal cavity	Abdominal wall	None	/
17	F	31	Squamous cell carcinoma (cervix)	8	PD	LN	NL (lung)	None	/
18	M	71	Adenocarcinoma (colorectum)	15	PD	lung	LN	PD-1	7.2
19	F	66	Adenocarcinoma (pancreas)	6	PD	peritoneal cavity	liver	None	/
20	F	42	Adenocarcinoma (liver)	12	SD	liver	LN	PD-1	28.2
21	F	43	Squamous cell carcinoma (cervix)	12	PD	LN	LN	PD-1+RT	24.5
22	F	37	Adenocarcinoma (Head and neck)	27	PR	lung	lung	PD-1	15.3

*F* Female, *M* Male, *LN* Lymph nodes, *NL* New lesions

### Survival analysis

The median OS for DR patients was 13.5 (95% CI 8.1–18.9) months. Regarding the other subgroups including uniform response, only stable lesions, and uniform progression, the median OS was 27.7 (95% CI 16.9–38.5), 17.7 (95% CI 14.4–21.0), and 5.9 (95% CI 4.0–7.8) months, respectively. Patients with DR had significantly longer OS than patients who showed a uniform progression (*P* = 0.012). There was no significant difference in OS between patients exhibiting a uniform response and those exhibiting only stable lesions (*P* = 0.285), or between patients exhibiting a uniform response and those exhibiting DR (*P* = 0.088). The uniform progression and DR are two different patterns of progression. After confirming progression, the overall survival of patients with DR was significantly longer than that of patients with uniform progression (9.9 months (95% CI 5.7–14.1) vs. 4.2 months (95% CI 1.9–6.5), *P* = 0.028).

**Fig. 3:**
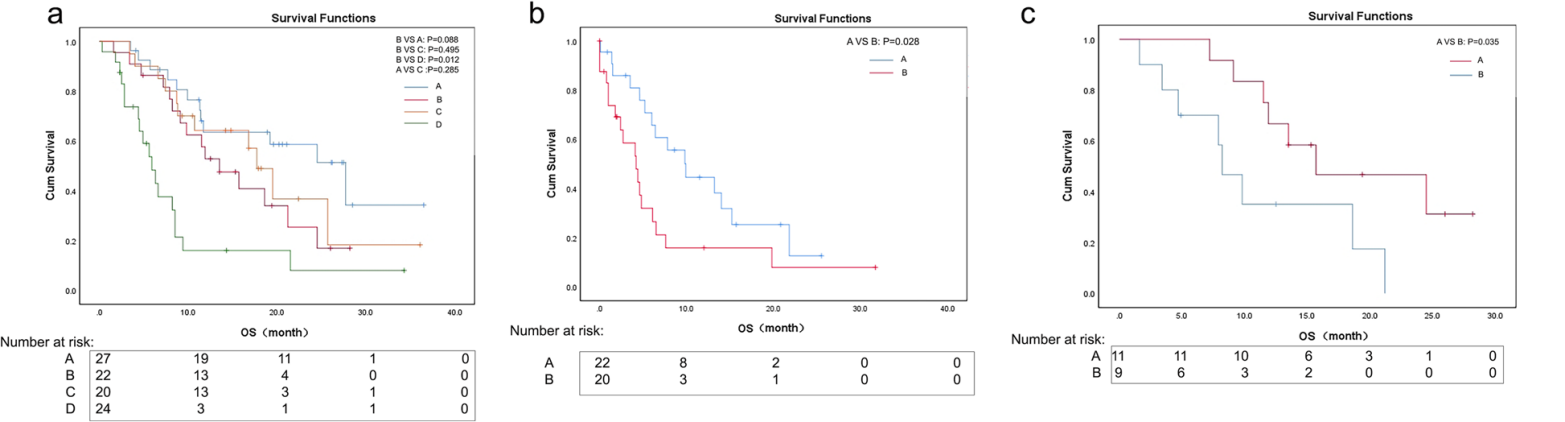
**a** Kaplan–Meier survival curves showing the overall survival stratified by tumor response. (A) Uniform response; (B) dissociated response; (C) only stable lesions; (D) uniform progression. **b** Kaplan–Meier curves of overall survival (OS) after confirming progression stratified by different patterns of progression. (A) Dissociated response; (B) uniform progression. **c** Kaplan–Meier curves of overall survival (OS) stratified by continued PD-1 inhibitors in combination with RT or PD-1 inhibitor monotherapy after DR diagnosis. (A) PD-1 + RT/PD-1 therapy after DR diagnosis; (B) non-PD-1 + RT/PD-1 therapy after DR diagnosis

Patients with DR who continued PD-1 inhibitors in combination with RT or PD-1 inhibitor monotherapy (*n* = 12) experienced significantly prolonged overall survival (15.7 (95% CI 3.5–27.9) vs. 8.2 (95% CI 5.6–10.8) months, *P* = 0.035) compared with patients who did not continue PD-1 inhibitors in combination with radiotherapy or PD-1 inhibitor monotherapy (*n* = 10) (Fig. 3a–c).

## Discussion

DR corresponds to mixed radiological or heterogeneous patterns of response at the same time point [22]. In this retrospective study, we analyzed the response of all measurable lesions to PD-1 inhibitors combined with RT in patients with advanced metastatic solid tumors at the initial CT/MRI. DR was not uncommon in our cohort, 23.7% (*n* = 22).

The incidence of this atypical reaction is unknown. Depending on the definition, the incidence of DR varied between 3.3 and 47.8% in different histological subtypes [23]. However, studies on DR in immunotherapy combined with radiotherapy are rare. Sun et al. [24] retrospectively analyzed the first follow-up CT images (median time: 2.8 months, IQR (2.0–3.4)) of six independent IORT clinical studies of patients with advanced solid tumors receiving immunotherapy combined with radiotherapy and found that 12.8% of patients presented with DR. In our study, the incidence of DR was significantly higher than the results of the study. We believe there are two reasons for this. First, the incidence of DR in our cohort was assessed at every follow-up image after treatment, rather than just at the first follow-up images. Second, the definition of DR is different. In addition to patients with both progressive lesions and responding lesions, patients with only responding lesions but with the appearance of new lesions or significant deterioration of unmeasurable lesions are also included in DR.

At the DR diagnosis, 2 patients showed RECIST-defined PR, 11 patients showed RECIST-defined SD, and 9 patients showed RECIST-defined PD in our study. The median OS of patients with DR was 13.5 months, which was significantly longer than that of patients with uniform progression (13.5 months vs. 5.9 months; *P* = 0.012). After confirming uniform progression and DR, the overall survival of patients with DR was also significantly higher than that of patients with uniform progression (9.9 months vs. 4.2 months, *P* = 0.028). Our results are consistent with previous studies [25] showing that RECIST 1.1 also does not adequately capture the kinetics and heterogeneity of the combination of an immune checkpoint inhibitor and radiotherapy. New criteria such as immune-related response criteria (irRC), immune-related RECIST (irRECIST), and immune RECIST (iRECIST) have been proposed to evaluate ICIs’ response and survival benefit [26–28]. However, these specific radiological criteria only target the PsPD and do not capture other response patterns, such as HPD and DR. Therefore, the future immunotherapy-adapted guidelines and criteria in solid cancer should not only solve the problem of PsPD but also solve the problem of atypical reactions such as DR.

The basic mechanism of DR is not clear. The combination of multiple factors may explain the underlying biological mechanism of the separation reaction. Firstly, genomic instability occurs during the clonal evolution of solid tumors, and different tumor clones may produce multiple coexisting metastases [29]. Secondly, microenvironment differences among metastases also cause heterogeneous responses [22]. Thirdly, tissue penetration differences of anticancer drugs may also be a potential cause. This pattern of response presents particular challenges for patient management. At present, there is no consensus on the clinical management of DR. Sato et al. [30] believe that the occurrence of DR does not always mean ICI resistance, and it should not be changed to other treatments prematurely. Humbert et al. [22] proposed that continuation of immunotherapy after DR can achieve a durable response. Zhou et al. [31] showed that patients with advanced NSCLC who continued ICI treatment post-DR derived apparent OS benefit than discontinuing counterpart. In our cohort, there was no specificity between the response and progressive sites in DR patients. 54.5% (12/22) of DR patients chose to continue PD-1 inhibitor combined with radiotherapy or PD-1 inhibitor monotherapy and experienced significantly prolonged overall survival compared with patients who did not continue. Therefore, it is a possible treatment option to continue ICI monotherapy or combined radiotherapy for DR patients with stable clinical conditions after comprehensively considering the degree of disease progression, patient status, and risk of immune-related adverse events.

Different from previous studies, women in our cohort had a significantly higher probability of developing DR than men (*P* = 0.018), which required us to verify in a larger sample. Our study may contribute to the development of appropriate management protocols for patients who develop DR during ICIs combined with radiotherapy. However, the study has some limitations. First, this is a retrospective study. Second, the heterogeneity of population and tumor may also lead to bias. Third, a small number of patients from a single institution were included. Therefore, a larger sample study is needed to further confirm the reliability of our results.

## Conclusion

DR is not uncommon in patients with advanced metastatic solid tumors treated with PD-1 inhibitors combined with radiotherapy and shows a relatively favorable prognosis. RECIST 1.1 does not adequately capture its dynamics and heterogeneity, which may underestimate the survival benefit in the combination treatment population. In patients who present with DR, continuing with a PD-1 inhibitor in combination with radiotherapy or PD-1 inhibitor monotherapy may be beneficial and may enable patients to achieve longer OS.

## Data Availability

The datasets used and/or analyzed during the current study are available from the corresponding author on reasonable request.

## Abbreviations

PD-1: Programmed cell death-1
PD-L1: Programmed cell death ligand-1
RT: Radiotherapy
DR: Dissociated response
OS: Overall survival
ICIs: Immune checkpoint inhibitors
CTLA-4: Anti-cytotoxic T-lymphocyte antigen-4
PsPD: Pseudo-progressive disease
DeR: Delayed response
HPD: Hyperprogressive disease
FastPD: Fast progressive disease
RECIST 1.1: The Response Evaluation Criteria in Solid Tumours v 1.1 guidelines
SBRT: Stereotactic body radiotherapy
HFRT: Hypofractionated radiotherapy
ECOG ps: The Eastern Cooperative Oncology Group performance status
CT: Computed tomography
MRI: Magnetic resonance imaging
EMR: The electronic medical record
IQR: Interquartile range
IORT: Radiotherapy with immunooncology
PR: Partial response
SD: Stable disease
PD: Progressive disease
irRC: Immune-related response criteria
irRECIST: Immune-related RECIST
iRECIST: Immune RECIST
NSCLC: Non-small cell lung cancer
